# Proteomic Changes of Activated Hepatic Stellate Cells

**DOI:** 10.3390/ijms222312782

**Published:** 2021-11-26

**Authors:** Maximilian Schinagl, Tamara Tomin, Juergen Gindlhuber, Sophie Honeder, Raphael Pfleger, Matthias Schittmayer, Michael Trauner, Ruth Birner-Gruenberger

**Affiliations:** 1 Institute of Chemical Technologies and Analytics, Technische Universität Wien, 1060 Vienna, Austria; maximilian.schinagl@tuwien.ac.at (M.S.); tamara.tomin@tuwien.ac.at (T.T.); raphael.pfleger@tuwien.ac.at (R.P.); matthias.schittmayer@tuwien.ac.at (M.S.); 2 Department of Pathology, Medical University of Graz, 8010 Graz, Austria; juergen.gindlhuber@medunigraz.at (J.G.); sophie.honeder@medunigraz.at (S.H.); 3 Hans Popper Laboratory of Molecular Hepatology, Division of Gastroenterology and Hepatology, Department of Internal Medicine III, Medical University of Vienna, 1090 Vienna, Austria; michael.trauner@meduniwien.ac.at

**Keywords:** hepatic stellate cells, activation, fibrosis, proteomics

## Abstract

Hepatic stellate cells (HSC) are the major cellular drivers of liver fibrosis. Upon liver inflammation caused by a broad range of insults including non-alcoholic fatty liver, HSC transform from a quiescent into a proliferating, fibrotic phenotype. Although much is known about the pathophysiology of this process, exact cellular processes which occur in HSC and enable this transformation remain yet to be elucidated. In order to investigate this HSC transformation, we employed a simple, yet reliable model of HSC activation via an increase in growth medium serum concentration (serum activation). For that purpose, immortalized human LX-2 HSC were exposed to either 1% or 10% fetal bovine serum (FBS). Resulting quiescent (1% FBS) and activated (10% FBS) LX-2 cells were then subjected to in-depth mass spectrometry-based proteomics analysis as well as comprehensive phenotyping. Protein network analysis of activated LX-2 cells revealed an increase in the production of ribosomal proteins and proteins related to cell cycle control and migration, resulting in higher proliferation and faster migration phenotypes. Interestingly, we also observed a decrease in the expression of cholesterol and fatty acid biosynthesis proteins in accordance with a concomitant loss of cytosolic lipid droplets during activation. Overall, this work provides an update on HSC activation characteristics using contemporary proteomic and bioinformatic analyses and presents an accessible model for HSC activation. Data are available via ProteomeXchange with identifier PXD029121.

## 1. Introduction

Liver fibrosis is the replacement of parenchymal liver tissue by non-parenchymal scar tissue [1]. This process mostly occurs due to liver inflammation from alcohol abuse [2], viral infection [3], or a non-alcoholic fatty liver [4]. In early stages, liver fibrosis is reversible upon treatment of the underlying etiology [5]. If left untreated liver fibrosis can progress to more severe conditions like cirrhosis and liver cancer [6]. However, there is still no approved treatment for the progression of fibrosis other than the removal of the source of inflammation [7].

Most fibrotic tissue generation can be attributed to a specialized cell type in the liver, the hepatic stellate cells (HSC) [8]. Under healthy liver conditions, these non-parenchymal cells merely act as a storage buffer for retinol (vitamin A), which is deposited as retinylesters in cytosolic lipid droplets (LD) [9]. Upon sustained liver inflammation, HSC transform from a quiescent (resting) phenotype towards a myofibroblast-like phenotype [1]. This transformation, termed “activation”, is a key event in liver fibrosis [10]. HSC display distinct characteristic changes during activation, which allows them to proliferate at an increased rate [11], migrate [12] towards the site of liver damage, lose their LD and increase their production of fibrotic proteins [13]. If we can improve our understanding of the cellular and molecular processes involved in HSC activation, we might identify targets to revert HSC to their quiescent phenotype, therefore alleviating fibrosis in a damaged liver.

Immortalized human HSC cell lines, which closely reflect primary HSC in vivo, are widely available [14]. These cell lines allow for accessible in-depth mechanistic investigations without the use of cancer cell lines or animal models. Often these HSC cell lines are treated with CCl_4_ or TGF-β to induce activation in vitro [15]. We chose a well described HSC cell line developed in 2005, termed Lieming Xu 2 (LX-2) [16], which displays a low activation (quiescent) state under low serum conditions [17]. The addition of serum activates LX-2 cells [17,18,19], making it an accessible model for studying HSC activation without the need for CCl_4_ or TGF-β. It is noteworthy to add that LX-2 cells never achieve a fully quiescent state when grown on plastic dishes [16].

Using several in vitro assays combined with proteomic and ontological characterization of LX-2 cells, we here report an in-depth analysis of serum LX-2 activation. We demonstrate that the phenotype of serum activated LX-2 cells is in line with other activation techniques and provide a comprehensive overview of changes that occur on protein expression level during the process of activation. Lastly, we highlight the underlying metabolic changes during HSC activation, such as deregulation of lipid and cholesterol metabolism.

## 2. Results

### 2.1. Hepatic Stellate Cells Are Activated by Fetal Bovine Serum

The LX-2 cell line shows characteristic features of primary HSC in vivo and displays a quiescent phenotype in growth medium with low serum concentrations but gets activated in high serum concentrations [16]. We here employed this easy and robust approach to study changes between quiescent and activated LX-2 cells.

**Figure 1:**
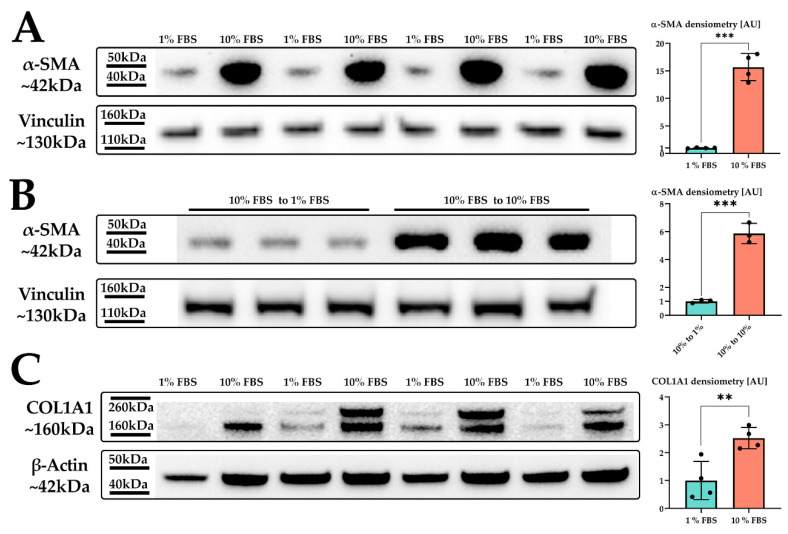
LX-2 HSC activation by serum. (**A**) α-SMA protein expression of LX-2 cells under low (1% FBS) or high (10% FBS) serum concentration. Vinculin was used as loading control. (**B**) α-SMA protein expression of LX-2 cells incubated in 10% FBS for 24 h, then for additional 24 h in either 1% FBS (10% FBS to 1 % FBS) or 10% FBS (10% FBS to 10% FBS). Vinculin was used as loading control. (**C**) COL1A1 protein expression of LX-2 cells under low (1% FBS) or high (10% FBS) serum concentration. One additional band can be seen at ~200 kDa, which was not used for quantification. Loading control was β-actin. ** *p* < 0.01; *** *p* < 0.001, unpaired *t*-test, two-sided.

We treated LX-2 cells with either 1% or 10% FBS for 24 h. Their activation status was confirmed by investigation of α-smooth muscle actin (α-SMA; Figure 1), a protein highly expressed in activated HSC which enhances the contractility of cells [20] and has been prominently featured as a reliable marker for HSC activation [20,21,22].

Indeed, we observed a strong upregulation in α-SMA protein expression upon increased serum concentration in the growth medium (Figure 1A). We next investigated whether the serum activation of LX-2 cells was reversible. For this, we incubated LX-2 cells in 10% FBS containing DMEM for 24 h, replaced the media with 1% FBS containing DMEM for additional 24 h and subsequently analyzed the expression of α-SMA. Figure 1B suggests that LX-2 cells can switch back from their activated to their quiescent state, depending on the serum concentration in the medium. In addition to α-SMA, activated HSC produce increased amounts of extracellular matrix (ECM) proteins, such as collagen [23,24]. Collagen 1 α1 (COL1A1) is frequently used as a marker for fibrosis [25] and therefore can also be used to indicate the activation status of HSC. Correspondingly, LX-2 cells indeed showed increased deposition of COL1A1 protein upon serum activation for 24 h (Figure 1C).

### 2.2. Serum Activated Hepatic Stellate Cells Show a Phenotype Characteristic of Activated Hepatic Stellate Cells

**Figure 2:**
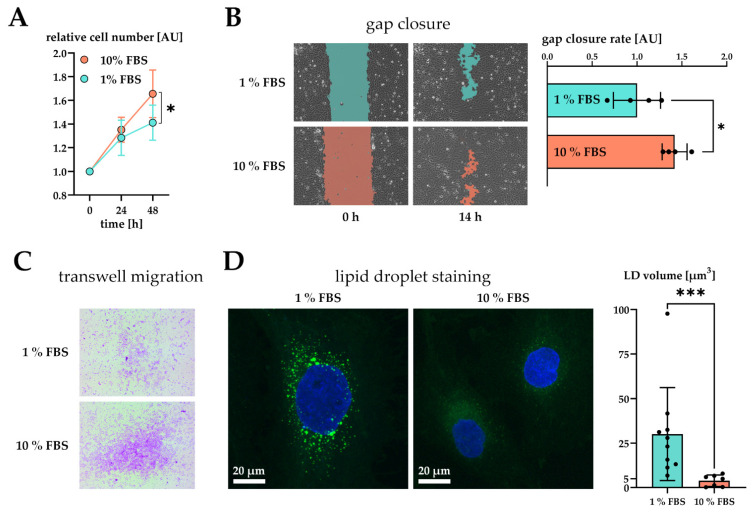
Effect of serum activation on LX-2 cell phenotype. (**A**) Relative proliferation of LX-2 cells under low (1% FBS) or high (10% FBS) serum concentrations, normalized to 0 h. (**B**) Left: gap closure assay of LX-2 cells in either 1% FBS or 10% FBS after 0 h and 14 h. Right: Quantification of gap closure rate where the increase of occupied space is measured against time and plotted as slope (normalized to 1 % FBS). Higher values represent faster migration. (**C**) Transwell migration of LX-2 cells through a membrane in either 1% FBS or 10% FBS after 24 h. These images show the underside of the membrane and therefore only migrated cells stained purple. (**D**) Left, both images: lipid droplets (BOPIPY, green) and nuclei (DAPI, blue) staining of LX-2 cells shown as maximum intensity Z-projection. Right: Quantification of lipid droplet volume per cell. * *p* < 0.05; *** *p* < 0.001, unpaired *t*-test, two-sided.

We next analyzed the phenotype of activated LX-2 cells. During activation, HSC transform into a myofibroblast-like phenotype, which is accompanied by an increased growth rate [11], enhanced migration [12], and the loss of LD volume [26]. We saw enhanced growth of LX-2 cells upon an increase in serum concentration after 48 h (Figure 2A).

Furthermore, we tested the migratory properties of activated LX-2 cells. We first performed a gap closure assay (scratch assay) where LX-2 cells must migrate in order to close a gap between confluent cell batches. To suppress serum-driven proliferation effects, we pre-incubated both conditions in 1% FBS and replaced the growth media by fresh 1% or 10% FBS containing DMEM at timepoint 0 h. We then monitored migration of cells for the period of only 14 h during which no growth difference was observed (Figure 2A). The difference in growth was prominent only after 48 h in culture (Figure 1A). As expected, serum activated LX-2 cells migrated faster, leading to quicker gap closure (Figure 2B). Faster migration was further corroborated by performing a transwell migration assay. For this purpose, LX-2 cells were seeded onto porous transwells at 1% or 10% FBS. Serum activated LX-2 cells showed enhanced migration to the lower side of the membrane after 24 h (Figure 2C).

We also investigated whether serum activation of LX-2 cells was accompanied by loss of LD. In order to induce LD formation, LX-2 were first incubated in 200 µM oleic acid at 1% FBS for 24 h and then treated with either 1% or 10% FBS containing DMEM for additional 48 h. We observed a decline in LD volume in serum activated LX-2 cells (Figure 2D). Furthermore, LD in serum activated LX-2 cells appeared to be smaller in accordance with [27].

Altogether, these findings confirm serum activation of LX-2 cells as an accessible option for studying the activated phenotype of HSC in vitro.

### 2.3. Proteomic Analysis of Serum Activated Hepatic Stellate Cells Reveals Changes of Several Key Cellular Pathways

To reveal global changes on protein level induced by HSC activation we examined the proteome of serum activated LX-2 cells. After treatment of LX-2 cells with either 1% or 10% FBS for 48 h, cells were harvested and subjected to shotgun proteomics. As a result, we quantified 4598 proteins across six biological replicates per treatment group (quiescent or activated). The protein list was filtered to keep only those proteins quantified in all six replicates in at least one of the treatment groups. The resulting protein matrix comprised of 3163 proteins (Supplementary Table S1) was subjected to statistical analysis. A Pearson correlation coefficient heatmap of our six replicates can be seen in Supplementary Figure S1.

**Figure 3:**
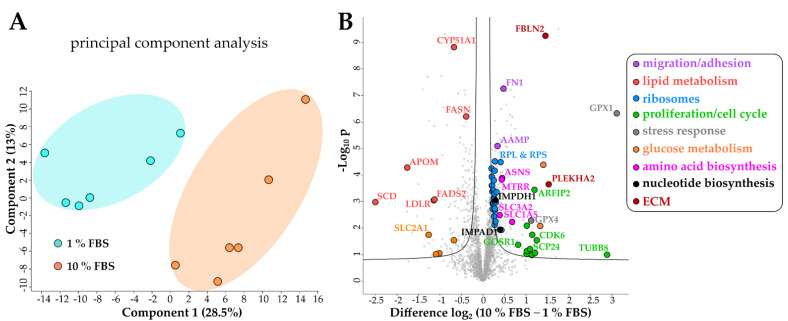
Changes of the LX-2 HSC proteome upon serum activation. (**A**) Principal component analysis shows a clear separation between serum activated (10% FBS) vs. quiescent (1% FBS) LX-2 cells. (**B**) Volcano blot of serum activated (10% FBS) vs. quiescent (1% FBS) LX-2 cells at FDR = 0.05 and S0 = 0.1. FDR corrected Student *t*-test *p*-value < 0.05, N = 6 biological replicates per group.

Principal component analysis (PCA) of the proteomics dataset revealed a clear separation between serum activated and quiescent LX-2 cells (Figure 3A). Correspondingly, as shown in the volcano blot in Figure 3B, serum activated LX-2 cells undergo a massive change to their proteome during activation; in numbers: 465 proteins were highly significantly up- or downregulated (two-sample *t*-test, two-sided, S0 = 0.1, permutation-based false discovery rate (FDR) = 0.05, 250 randomizations). In accordance with the observed phenotypes, activated stellate cells had a higher abundance of proteins involved in migration (e.g., fibronectin 1 FN1, angio-associated migratory cell protein AAMP, Figure 3B, marked in purple), reduced levels of proteins involved in lipid biosynthesis (e.g., FASN, CYP51A1, Figure 3B, marked in red) and a prominent up-regulation of ribosomal proteins (Figure 3B, marked in blue).

#### 2.3.1. Serum Activated Hepatic Stellate Cells Show Increased Ribosome Biogenesis, Cell Cycle, Cell Migration and Oxidative Stress Related Proteins

**Figure 4:**
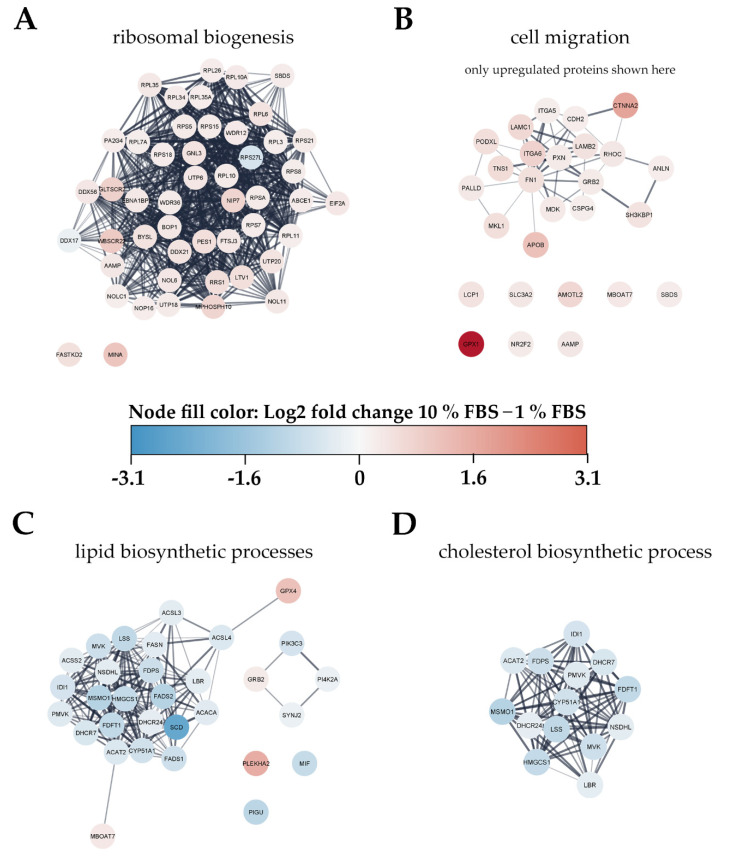
Visualization of changes to the proteome of LX-2 cells during serum activation. The color bar in the center indicates up- (red) or downregulated (blue) proteins in serum activated (10% FBS vs. 1% FBS) LX-2 cells. (**A**) Ribosomal proteins (small ribosomal subunits RPS and large ribosomal subunits RPL) as well as proteins related to ribosomal biogenesis were upregulated in serum activated LX-2 cells. (**B**) Many proteins related to cell migration like AAMP and FN1 are upregulated in serum activated LX-2 cells fitting the observed increase in cell migration (Figure 2B,C). (**C**) Serum activated LX-2 cells show a decreased expression of proteins involved in the lipid biosynthetic process fitting the observed decrease in LD volume (Figure 2D). (**D**) Supplementary to (**C**), proteins involved in cholesterol biosynthesis are distinctly downregulated in serum activated LX-2 cells.

To gain an overview of metabolic pathways and protein classes affected by serum activation we used significantly changed proteins as input for protein network and functional enrichment analysis using the STRING [28] database for analysis and Cytoscape [29] for visualization (Figure 4). As one of the most prominently enriched clusters in serum activated LX-2 cells we identified ribosome biogenesis related proteins, including small ribosomal proteins (RPS) as well as large ribosomal subunit proteins (RPL) (gene ontology (GO) process ribosome biogenesis GO:0042254, 49 enriched genes out of 270 pathway genes, FDR: 3.2 × 10^−4^ for proteins with enrichment values, enrichment score: 1.02665; Figure 4A). Additionally, we identified 32 proteins involved in rRNA processing (GO:0006364), of which 31 were upregulated in serum activated cells (Supplementary Figure S2A).

Furthermore, we also observed increased expression of Golgi-ER traffic related proteins (Tubulin beta-8 chain TUBB8, Arfaptin-2 ARFIP2, Golgi SNAP receptor complex member 1 GOSR1; see Supplementary Table S2). These findings are in line with the observation of increased ECM protein production (see Figure 1C).

Additionally, upregulated proteins in serum activated LX-2 cells like cell division protein kinase 6 (CDK6) or kinetochore protein Spc24 (SPC24) imply an upregulation of the cell cycle (Figure 3B, green dots), which can also be seen in Figure 2A where we show increased proliferation in serum activated LX-2 cells. In line with these findings, we found upregulated nucleotide biosynthesis proteins in activated HSC (Figure 3B, black dots).

Interestingly, proteins related to cellular stress response like glutathione peroxidase 1 (GPX1), and GPX4 are highly upregulated in serum activated LX-2 cells (Figure 3B, grey). Glutathione peroxidases were shown to reduce oxidative stress in HSC [30].

Proteomic network analysis also suggested increased expression of proteins related to cell migration, including the aforementioned AAMP and FN1 in serum activated LX-2 cells. Although STRING functional enrichment analysis with values/ranks did not identify cell migration to be significantly enriched, 27 out of 37 matched proteins involved in this GO term were found significantly upregulated. (Figure 4B; cell migration GO process GO:0016477, 37 enriched genes out of 812 pathway genes, FDR: 0.0151 for upregulated proteins only, network strength: 0.35). This observation is in line with the phenotype we describe in chapter 2.1.1 and Figure 2B,C.

#### 2.3.2. Activated Hepatic Stellate Cells Show a Decrease in Fatty Acid and Cholesterol Biosynthesis

Interestingly, lipid biosynthetic processes were found to be downregulated in serum activated LX-2 cells (GO process GO:0008610, 30 enriched genes out of 585 pathway genes, FDR: 2.8 × 10^−4^ for proteins with enrichment values, enrichment score: 2.08448), including all fatty acid desaturases (SCD (being the most downregulated protein (6-fold)), FADS1, FADS2), acetyl-CoA-carboxylase 1 (ACACA), and fatty acid synthase (FASN; Figure 4C). Some of these findings are in accordance with previous reports as FASN downregulation was already reported on mRNA level in activated (10 % FBS) as compared to quiescent (2 % FBS) LX-2 cells [18]. These observations are in accordance with the LD volume changes described in chapter 2.1.1 and Figure 2D. However, not only fatty acid de-novo synthesis and desaturation were affected during activation. Cholesterol biosynthesis was another prominently downregulated pathway in activated HSC (GO process GO:0006695, 14 enriched genes out of 41 pathway genes, FDR: 7.4 × 10^−3^ for proteins with enrichment values, enrichment score: 2.62406). In addition, lipid transport proteins were reduced, e.g., apolipoprotein M (APOM) and low-density lipoprotein receptor (LDLR), see Figure 3B, red dots.

**Figure A1:**
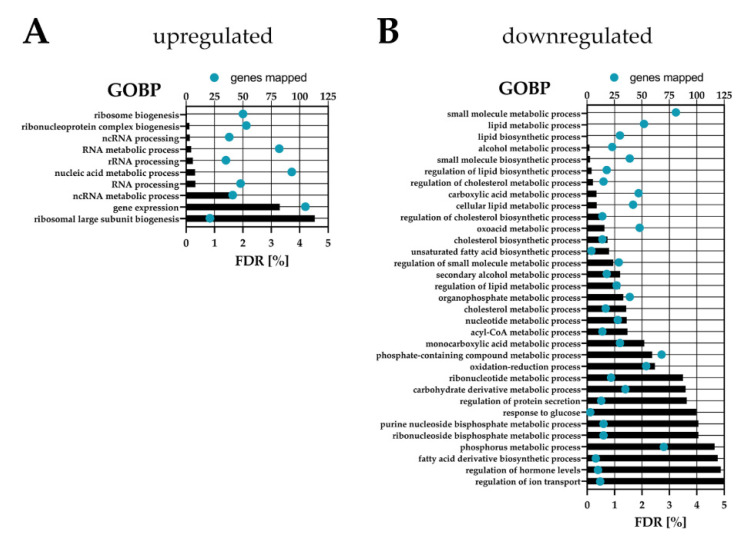
STRING gene-ontology-enrichment analysis of biological processes (GOBP) of significantly upregulated (**A**) or downregulated (**B**) proteins in activated LX-2 cells. Blue dots represent the number of genes found in the corresponding pathway on the left.

Overall, the proteomic alterations suggest several metabolic changes. Additionally, we identified a downregulation of glucose transporter SLC2A1 in serum activated LX-2 cells, see Figure 3B, yellow dots, suggesting decreased glucose uptake. Lastly, we also detected changes in abundance of several ECM proteins (Supplementary Table S1). However, we could not identify upregulation of COL1A1 like we see in our western blot (Figure 1C) or a clear trend in the expression of other collagen proteins in our proteomics dataset (Supplementary Table S1). One reason for this might be the proteomics sample preparation: while for western blot analysis trypsin was used for cell harvest, for proteomics analysis physical detachment using cell scrapers was applied. This might suggest that cell scraping does not assure a quantitative collection of collagen proteins. Additionally, crosslinked ECM proteins with low solubility are notoriously difficult to quantify using proteomics [31]. Despite this, we could identify many higher expressed regulators of ECM production, including fibulin-2 (FBLN2) and pleckstrin homology domain-containing family A member 2 (PLEKHA2) in serum activated LX-2 cells (Figure 3B, dark red dots). Additional STRING gene-ontology-enrichment analysis results are summarized in Figure A1.

## 3. Discussion

Activation of HSC is a well described phenomenon and key event in liver fibrosis [8]. This transformation process involves major changes to the phenotype and the proteome of the cells. In this study, we describe a robust and accessible model of HSC activation and characterize it in depth by phenotypic, proteomic, and gene ontology enrichment analyses. Combined, our data confirms and adds on observations in activated HSC in vivo and in vitro and suggests additional pathways involved in or affected by HSC activation.

Even though serum activation of human LX-2 cells has been used a number of times before [18,19,32,33,34,35] it has not yet been characterized in depth with regard to changes in gene and protein expression compared to rat HSC serum activation [36]. Therefore, we first confirmed that the addition of FBS to growth media is both an easy and reliable way to activate LX-2 cells. Both α-SMA, as well as COL1A1 expression changes suggested prominent activation of LX-2 cells under high serum conditions (Figure 1A,C). Additionally, LX-2 serum activation appeared to be reversible (Figure 1B). 

We further characterized serum activated LX-2 cells by investigating several prominent phenotypical changes described previously. Activated HSC have been reported to show increased proliferation rates similar to myofibroblasts during wound healing [11]. We confirm this property: Serum activated LX-2 cells proliferated significantly faster after 48 h in culture (Figure 2A). During activation, HSC also have been shown to gain enhanced migratory properties helping them move towards the site of injury [12]. We also confirm this observation using both gap closure (Figure 2B) and transwell (Figure 2C) migration tests with serum activated LX-2 cells migrating significantly faster in both cases. Lastly, since HSC have been described as losing most of their cytosolic LD during activation [26], we compared the cellular LD volume of serum activated vs. quiescent LX-2 cells. Serum activated LX-2 cells indeed lost most of their LD volume compared to quiescent LX-2 cells (Figure 2D). Taken together, the serum activated LX-2 cell model appears to provide an accurate representation of the phenotype shown by activated HSC in vitro.

We further investigated changes to the proteome of the HSC during activation. Prior work on the activated HSC proteome revealed enhanced ECM organization in LX-2 cells [37] and increased proliferation and migration, as well as decreased lipid metabolism in activated rat HSC [38]. Our analysis showed that many of the phenotypical changes during activation are also represented in the proteome of LX-2 cells. We discovered enhanced ribosomal biogenesis in serum activated LX-2 cells (Figure 4A). This increased need for protein production has been identified in transcriptome analysis of activated rat HSC [39]. Interestingly, overexpression of 40S ribosomal protein S5 (RPS5) was found to suppress HSC activation in rat HSC [40], while we found RPS5 to be upregulated in serum activated LX-2 cells (Figure 4A). We hypothesize that the general increased need for the ribosomal machinery, as well as rRNA processing (Supplementary Figure S2A) is related to the higher rates of proliferation as well as remodeling of the proteome, e.g., additional production of ECM proteins (such as COL1A1; Figure 1C) and the general re-structuring of HSC during activation. Additionally, Golgi-ER traffic related proteins are enriched (Supplementary Table S2) indicating increased protein transport and secretion. 

Furthermore, many proteins involved in migration were also found to be upregulated in serum activated LX-2 cells (Figure 4B), including FN1, which has been reported to promote migration in HSC [41], as well as AAMP, which is mainly expressed in cells with a migratory phenotype [42]. These two findings, combined with many other upregulated proteins involved in migration (see Figure 4D, Supplementary Table S1) and our phenotyping experiments (Figure 2B,C) paint a clear picture of enhanced migration in serum activated LX-2 cells.

Next, we took a closer look at proteins involved in lipid metabolism since we already saw a decrease in LD volume in serum activated LX-2 cells. In our STRING protein network analysis we did not find increased lipid catabolism per se, other than the slight upregulation of carnitine-pamitoyltransferase 1 CPT1 (Figure 4C, Supplementary Table S1), but a decrease in lipid biosynthesis (Figure 4C), which might explain the loss of LD volume during activation (Figure 2D). Chen et al., 2007 showed that activated rat HSC exhibit a decrease in lipogenic genes [43], which was similar to our results. Furthermore, they reported increasing numbers of mitochondria in activated rat HSC. In our experiments, we could not identify an upregulation of mitochondrial proteins, which would hint at an increase in β-oxidation and the capacity for oxidative phosphorylation during activation, but rather a decrease of mitochondrial matrix proteins (Supplementary Figure S2B) in serum activated LX-2 cells. Even though serum activated LX-2 cells are exposed to higher concentrations of fatty acids and lipoproteins in their growth medium, they do not upregulate the expression of mitochondrial proteins to increase β-oxidation. Although activated HSC have been reported to preferentially utilize aerobic glycolysis [43] serum activated LX-2 cells downregulated the glucose transporter SLC2A1 (Figure 3B) in the provided high-glucose (4.5 g/L) growth medium.

Contrary to the observed reduction of glucose transporter and lipid metabolic enzymes we could identify upregulation of several amino acid biosynthetic proteins (asparagine synthetase ASNS, methionine synthase reductase MTRR) and transporters (neutral amino acid transporter SLC1A5, 4F2 cell-surface antigen heavy chain SLC3A2) in serum activated LX-2 cells (Figure 3B). We suggest LX-2 cells upregulate these pathways to enable the increased ribosome and ECM protein production during serum activation.

Bhattacharya et al. [44] reported downregulation or inhibition of stearoyl CoA-desaturase SCD in human HSC ameliorates fibrosis by a decrease in cholesterol biosynthesis. In our work we show that SCD and cholesterol biosynthesis in general are vastly downregulated in serum activated LX-2 cells (Figure 4D). We suggest that this downregulation can in part be explained by the increased availability of cholesterol from FBS in serum activated LX-2 cells, reducing the activation of sterol regulatory element-binding proteins SREBPs (Supplementary Table S2). An overview of observed phenotypical changes during serum activation of LX-cells in comparison to literature can be found in Table 1.

**Figure A2:**
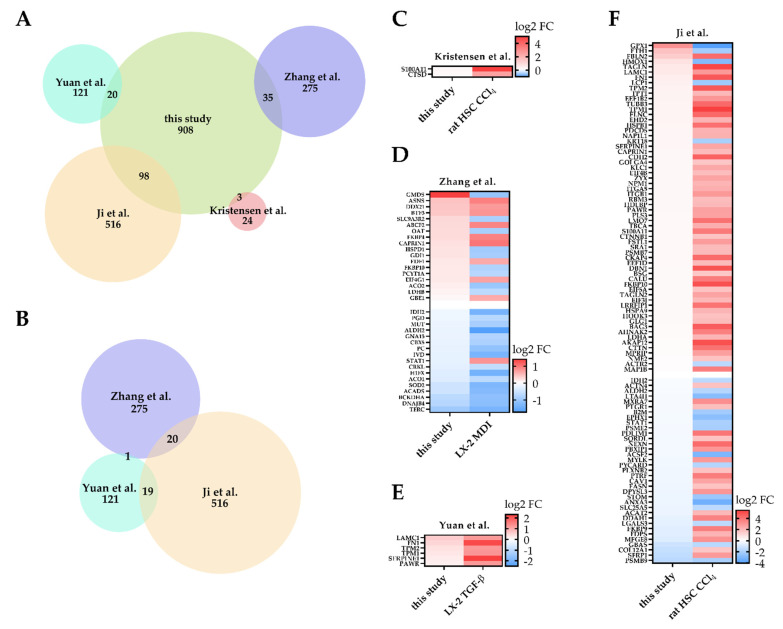
Comparison of this study to previous works of HSC proteomic analysis. In this figure we used all identified proteins with a *p*-value < 0.05. Images (**C**–**F**) compare the log2 fold change (FC) transformed activated HSC vs. quiescent HSC. (**A**) Venn diagram of overlapping proteins found in this study, compared to other proteomic studies. Numbers indicate identified proteins. (**B**) Venn diagram comparing differentially regulated proteins of previous studies with each other. The study by Kristensen et al. was excluded due to low quantified protein numbers. (**C**) Comparing proteins in in vivo CCl_4_ activated rat HSC by Kristensen et al. [49] to our proteomics dataset. We excluded Farnesyl pyrophosphate synthetase (FDPS) in the comparison, due to missing values for quiescent HSC in the Kristensen et al. dataset. (**D**) Serum activated LX-2 cells vs. MDI adipocyte differentiation mixture reverted LX-2 cells [50] are compared to serum activated LX-2 cell proteome. (**E**) LX-2 cells activated with TGF-β [37] are compared to serum activated LX-2 cell proteome. (**F**) In vivo CCl_4_ activated rat HSC [38] are compared to serum activated LX-2 cell proteome.

Lastly, we compared our serum activated LX-2 proteome to previous works on the activation of HSC. Here, we analyzed proteomic studies from CCl_4_ in vivo activated rat HSC [38,49], as well as LX-2 cells activated with either TGF-β [37] or the reversion of FBS activated LX-2 cells with the adipocyte differentiation mixture MDI (isobutylmethylxanthine, dexamethazone, and insulin) [50]. A comparison of differentially regulated proteins found in both this study and the aforementioned previous works is displayed in the Appendix A Figure A2. In Figure A2A, a Venn diagram is displayed depicting differentially regulated proteins found in both literature and this study. The expression levels of these proteins are compared in Figure A2C–F. Additionally, proteins overlapping from different studies are visualized in Figure A2B. Figure A2C displays a heatmap comparing differentially regulated proteins found in CCl_4_ rat activated HSC to differentially regulated proteins found in serum activated LX-2 cells in this study. Interestingly, in this study, Kristensen et al. (Figure A2C) as well as Ji et al. (Figure A2F) identify the upregulation of the calcium-binding protein S100A11 in activated HSC. The reversion of serum activated LX-2 cells with MDI is compared to serum activated LX-2 cells in Figure A2D. Here we compare proteins differentially regulated in activated LX-2 cells. This comparison shows that especially proteins downregulated in both datasets match to a high extent. Interestingly, STAT1, which was identified as a key regulator in the reversion of LX-2 cells [50] was downregulated in our dataset, but strongly upregulated in the dataset of Zhang et al. However, STAT1 was also found to be downregulated in activated rat HSC cells (Figure A2F [38]), providing no clear hypothesis for the regulation of this protein. Furthermore, the amino acid biosynthetic protein ASNS was found to be upregulated in both datasets, solidifying the role of increased amino acid biosynthesis in activated HSC. The activation of LX-2 cells using TGF-β by Yuan et al. exhibits many similarities to our study when we compare differentially regulated proteins (Figure A2E). Surprisingly, most differentially regulated proteins found in both studies are involved in cell migration, like Laminin subunit gamma-1 (LAMC1), FN1, Tropomyosin alpha-1 chain (TPM1) and TPM2, as well as Plasminogen activator inhibitor 1 (SERPINE1), which are all upregulated in both studies (all these proteins are also upregulated in the dataset of Ji et al.). This is another hint at increased migration in activated LX-2 cells. Furthermore, PRKC apoptosis WT1 regulator protein (PAWR), a pro-apoptotic protein is upregulated in activated HSC in this study, as well as in the dataset of Yuan et al. and Ji et al. Figure A2F compares differentially regulated proteins in CCl_4_ activated rat HSC to differentially regulated proteins in serum activated LX-2 cells from this study. This comparison highlights especially the similarity in upregulated proteins in activated HSC. The ECM proteins FBLN2 as well as FN1 are both upregulated in this study, as well as in the dataset of Ji et al. Even though GPX1 and 4 are upregulated in (Figure 3B) in serum activated LX-2 cells in this study, Ji et al. suggests a downregulation of GPX1 in CCl_4_ activated rat HSC, which has also been shown by Hamid et al. [51]. Conversely, TGF-β activation of LX-2 cells increases GPX4 expression [52]. Furthermore, activated primary rat HSC also increase their expression of GPx over time [30]. Therefore, we suggest that the expression of GPx might differ between different activation methods.

In conclusion, we show that serum activated LX-2 cells exhibit a phenotype representative of HSC in vitro. We present serum activation of LX-2 cells as a viable alternative to other activation techniques and describe proteomic changes during the activation of HSC in accordance with the observed phenotypic changes of enhanced proliferation and migration and decreased lipid storage. Finally, we compare previous proteomic studies on HSC activation to this study.

## 4. Materials and Methods

### 4.1. Cell Culture

LX-2 cells (EP-CL-0560, Szabo Scandic, Vienna, Austria) were cultured in high-glucose Dulbecco’s Modified Eagle Medium (DMEM) from Sigma-Aldrich supplemented with 2 mM L-Glutamine and either 1 or 10 % FBS (Gibco) in plastic dishes (VWR, Radnor, Pennsylvania, United States). For all assays we used 1% FBS DMEM, unless stated otherwise. Cells were maintained at 37 °C at 5% CO_2_ and 20% O_2_. LX-2 cells were subcultured every 72 h at a ratio of 1:5 and used for experiments after at least two passages of subculturing. Seeding equal numbers of cells was assured using the CASY cell counting system. LX-2 cells for western blot and proteomic analysis were harvested after 24 and 48 h, respectively.

### 4.2. Proliferation Assay

LX-2 cells were seeded in 96-well plates at 10,000 cells per well at 1% FBS containing DMEM growth medium. After 24 h, the medium was replaced by 100 µL of either 1% or 10% FBS containing DMEM. Cell proliferation was assessed every 24 h using the cell counting kit 8 assay (CCK 8, Sigma-Aldrich, St. Louis, MO, USA) according to protocol.

### 4.3. Western Blotting Analysis

LX-2 cells were harvested after 24 h incubation time at either 1 or 10 % FBS at ~85 % confluency using trypsin-EDTA. Cells were lysed in CST lysis buffer (Cell Signaling Technologies, Danvers, MA, USA) supplemented with protease inhibitor cocktail (Sigma Aldrich). SDS-PAGE (NuPage, Thermo Fisher, Waltham, MA, USA) was used to resolve equal amounts of protein. Semi-dry blotting was performed onto nitrocellulose membranes (Amersham, GE Healthcare, Chicago, IL, USA). Blocking of membranes was achieved by 5% skim milk in TBS-T for 30 min at RT. Primary antibody (α-SMA, Invitrogen 1A4; vinculin, Invitrogen 7F9) incubation was performed over night at 4 °C and secondary HRP antibody (horse anti-mouse 7076, Cell Signaling Technologies) incubation was performed at RT for 1 h. Chemiluminescent detection agent Pierce ECL PLUS (Thermo Fisher) was used as substrate and detection was done at a ChemiDoc MP (BioRad, Hercules, CA, USA). Image analysis was carried out with Image Lab (BioRad). 

### 4.4. Proteomic Analysis

LX-2 cells were harvested using cell scrapers after 48 h incubation at either 1% or 10% FBS at ~85% confluency. Cells were washed with PBS and lysed with an in-house reducing and alkylating buffer (100 mM TRIS HCl; pH = 8.5, 1% sodium dodecyl sulphate, 10 mM tris(2-carboxyethyl) phosphine, 40 mM 2-chloroacetamide). After sonication (1 kJ), samples were heated to 95 °C for 10 min. 100 µg of protein per sample (after bicinchoninic acid assay protein estimation (BCA), Thermo Fisher Scientific, reducing agent compatible) were subjected to acetone precipitation by adding NaCl to a final concentration of 10 mM. After that, 4× volumes of acetone were added and incubated for 10 min. After centrifugation (10 min at 14,000× *g*) the supernatant was removed. Dried samples were dissolved in 25% trifluoroethanol in 100 mM Tris-HCl (pH = 8.5) and subjected to sonication until completely dissolved. For protein digest, samples were diluted to 10% trifluoroethanol using 100 mM ammonium bicarbonate. LysC and trypsin (both Thermo Fisher Scientific) were added in a 1:100 enzyme to protein ratio and digest was done overnight at 37 °C. Samples were desalted the following day using in-house prepared polystyrene-divinylbenzene, reversed-phase sulfonate stage tips (Supelco) and re-suspended in 0.1% formic acid.

Chromatography was carried out on an Ultimate 3000 RCS Nano Dionex system equipped with an Ionopticks Aurora Series UHPLC C18 column (250 mm × 75 µm, 1.6 µm). Separation was achieved via a linear gradient of H_2_O (solvent A) and acetonitrile, both with 0.1% formic acid added (solvent B) (0–5.5 min 2% B; 65.5 min 17% B; 95.5 min 25% B; 105 min 37% B; 115.5–125.5 min 95% B; 126-136.5 min 2% B) with a flow rate of 0.4 µL/min. Column temperature was kept at 40 °C.

Mass spectrometric analysis was performed on a timsTOF Pro (Bruker Daltonics, Billerica, Massachusetts, United States) in positive data dependent Parallel Accumulation-Serial Fragmentation (PASEF) [53] mode with enabled trapped Ion Mobility Spectrometry (TIMS) at 100% duty cycle (100 ms cycle time). Source capillary voltage was set to 1500 V and dry gas flow to 3 L/min at 180 °C. The proteomics data have been deposited to the ProteomeXchange Consortium via the PRIDE [54] partner repository with the dataset identifier PXD029121.

### 4.5. Proteomic Data Analysis

Data analysis, database search and protein quantification were performed with MaxQuant version 2.01.0 [55]; and statistical data analysis with Perseus version 1.6.14.0 [56]. Search criteria: false discovery rate (FDR) for peptide, peptide-to-spectrum as well as protein matches was set to 1%. Peptide tolerance was set to ±20 and ±4.5 for the first and main peptide search, respectively. Product mass tolerance was set to ±0.5 Da. Cysteine carbamidomethylation was set as static whereas methionine oxidation and N-terminal acetylation were set as dynamic modifications. Minimum required peptide length was six amino acids and maximum number of allowed tryptic mis-cleavages was two. For protein search, the SwissProt human FASTA file (downloaded on 30 November 2020 from https://www.uniprot.org, 20,434 entries) containing most common protein contaminants was used as a database. Label-free protein quantitation (LFQ) was performed with a minimum of two peptides per protein (unique and razor) as quantitation requirement. Match between runs was enabled in the retention time window of 1 min and alignment window of 20 min respectively. This resulted in a list of 4598 proteins with their corresponding LFQ values. LFQ values were log2 transformed (resulting in invalid values [NaN] for any missing value), and contaminants were removed. The matrix was then filtered to contain 100% of valid values (LFQ intensities > 0 prior transformation) in at least one group (1% or 10% FBS) to exclude proteins that were not identified across all samples of one group. This reduced the matrix to 3163 proteins, and remaining missing values were imputed from normal distribution (downshift 1.8, width 0.3). Consequently, two-sided student t-tests were performed with the following criteria: *p*-value of 0.05, S0 of 0.1 and permutation-based FDR set to 5% to correct for multi-testing with 250 randomizations. A full table of all proteins with values can be found in Supplementary Table S1. A volcano blot of protein expression differences was generated using identical parameters from the two-sided student t-tests mentioned earlier and can be found in Figure 3B. Principal component analysis was performed without category enrichment and can be found in Figure 3A. Histograms of data distribution per each sample is displayed in Supplementary Figure S3. Further data analysis and graphical interpretation of protein networks was performed using Cytoscape [29] v3.8.2. STRING [28] v11.5 was used to generate protein networks using the following parameters: Homo Sapiens, full STRING network, medium confidence (0.4), medium FDR stringency (0.05). Additional enriched pathways can be found in Supplementary Table S2.

### 4.6. Migration Gap Closure Assay

LX-2 cells were seeded in silicone 2-well inserts (ibidi, Gräfelfing, Germany) in 12-wells at 50,000 cells per silicone well in 100 µL 1% FBS containing DMEM. 2 mL of 1% or 10% FBS containing DMEM were placed outside the silicone insert. After 24 h, silicone inserts were carefully removed, and cells were imaged in 4 positions on a cell observer (Zeiss, Jena, Germany) at 37 °C in 20% O_2_ and 5% CO_2_ for 24 h. Gap closure was assessed by an in-house script by Juergen Gindlhuber in NIS-Elements v5.20.02 (Nikon, Tokyo, Japan).

### 4.7. Migration Transwell Assay

Transwell inserts (12-well, polycarbonate, 8 µm pore size) were bought from Thermo Scientific. LX-2 cells were seeded in 12-well transwell inserts and put into 12-wells containing either 1% or 10% FBS for 24 h. For microscopic analysis, inserts were removed from the 12-well plate, washed twice with PBS and formaldehyde fixed for 2 min at room temperature (RT). After that, cells were washed twice with PBS, permeabilized with methanol for 20 min at RT, stained with crystal violet for 15 min at RT and again washed twice with PBS. Finally, cells from the top layer of the transwell were carefully removed using a cotton swab. Before recording on an inverse microscope, transwells were placed onto a glass slide with the transwell membrane facing down. Additional images of Transwell migration can be found in Supplementary Figure S4.

### 4.8. Lipid Droplet Analysis

125,000 LX-2 cells were seeded onto glass cover slips (ROTH, 18 × 18 mm, thickness 0.13–0.16 mm) in 6-wells in 1% FBS media and after they were allowed to settle for 2 h treated with 200 µM oleic acid bovine serum albumin conjugate to initiate LD formation. After 24 h, the medium was replaced by either 1% or 10% FBS containing medium. After 48 h of additional incubation, LD were stained with BODIPY 493/503 (Invitrogen) for 10 min at 37 °C and then washed twice with pre-warmed PBS. After that, cells were fixed with 3.7% formaldehyde for 10 min at 37 °C and nuclei received DAPI staining (Vectashield, Vectalabs). Cover slips were placed onto glass slides (ROTH) and imaged on a Nikon A1 confocal microscope. LD volume was calculated using an in-house FIJI [57] script by Juergen Gindlhuber.

## Figures and Tables

**Table 1:** At a glance: Similarities and differences between literature and results presented in this study.

Phenotype	Model in Literature	Results in Literature	Results in This Study	References
Proliferation	Primary rat HSC	Increase of HSC numbers after CCl_4_ injury (activation)	Increased proliferation after serum activation, upregulation of ribosome biogenesis and cell cycle proteins	[45]
Migration	Primary rat HSC	Increased motility after PDGF-β activation	Increased migration after serum activation, upregulation of migration associated proteins	[46]
ECM production (collagen)	Primary rat HSC	Strong upregulation of collagen production in HSC CCl_4_ treatment (activation)	Increased COL1A1 production in serum activated LX-2, upregulation of ECM regulators and ER-Golgi transport	[47]
Lipid metabolism	Primary rat HSC	LDs increase in number, but decrease in total volume during activation	LDs decrease in volume, downregulation of proteins involved in lipid biosynthesis after serum activation	[27]
Cholesterol metabolism	Primary rat HSC, primary human HSC & LX-2	Downregulation of cholesterol biosynthesis in HSC ameliorates liver fibrosis	Downregulation of proteins involved in cholesterol biosynthesis in serum activated LX-2	[44,48]

## Data Availability

The mass spectrometry proteomics data have been deposited to the ProteomeXchange Consortium via the PRIDE [41] partner repository with the dataset identifier PXD029121.

## Supplementary Materials

The following supplements are available online at https://www.mdpi.com/article/10.3390/ijms222312782/s1.
