# Supplementary material for: Proteomic Changes of Activated Hepatic Stellate Cells
Source: Int J Mol Sci. 2021 Nov 26;22(23):12782. doi: 10.3390/ijms222312782 (PMC8657869; doi:10.3390/ijms222312782)
Supplement: Supplementary file 1 [file ijms-22-12782-s001.zip › ijms-1463714-SM proofed/ijms-1463714-supplementary for proofreading/supplementary data.pdf]

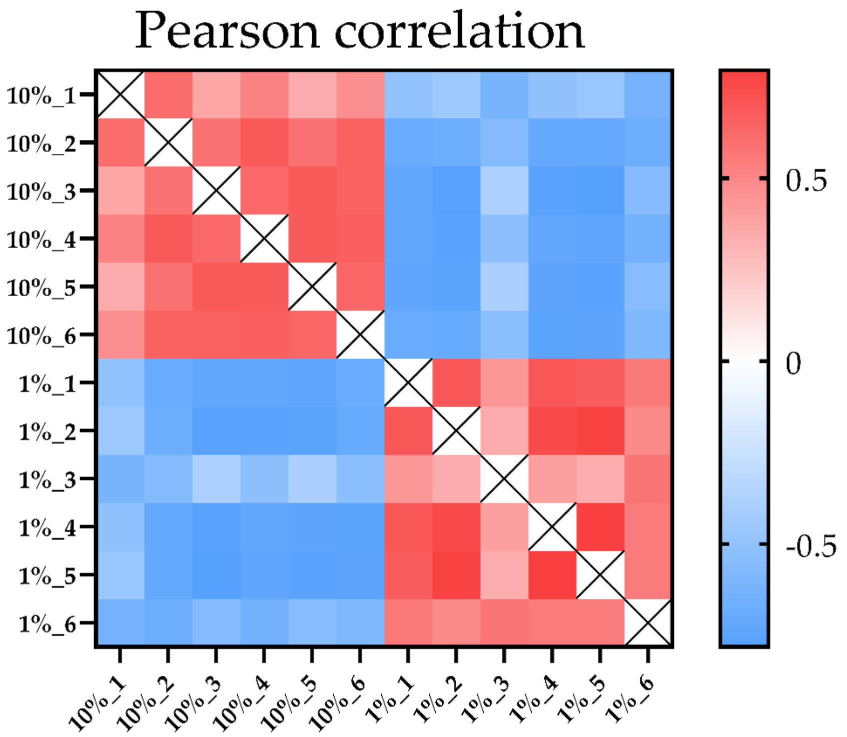

Figure S1. Pearson correlation of biological replicates for proteomic analysis (only proteins which passed two-sided student t-tests with the following criteria: p-value of 0.05, S0 of 0.1 and permutation-based FDR set to 5 % to correct for multi-testing with 250 randomizations). Numbers indicate percentage of FBS in growth media.

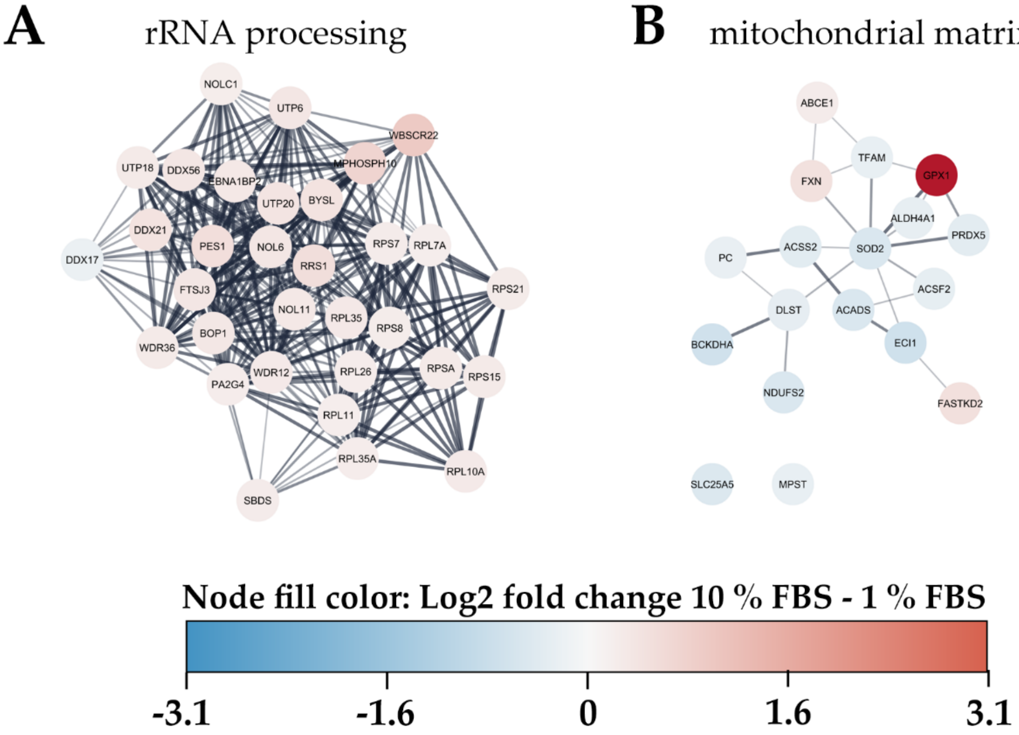

Figure S2. Additional visualization of changes to the proteome of LX-2 cells during serum activation. The color bar represent indicates up- (**red**) or downregulated (**blue**) proteins in serum activated (10 % FBS vs. 1 % FBS) LX-2 cells. (**A**) rRNA processing related proteins appear to be mostly upregulated in serum activated LX-2 cells. GO process rRNA processing GO:0006364 35 enriched genes out of 192 pathway genes, FDR: 0.0024 for proteins with enrichment values, enrichment score: 1.02948 (**B**) Proteins of the mitochondrial matrix are mostly downregulated in serum activated LX-2 cells. GO component mitochondrial matrix GO:0005759 22 enriched genes out of 463 pathway genes, FDR: 0.0143 for proteins without enrichment values.

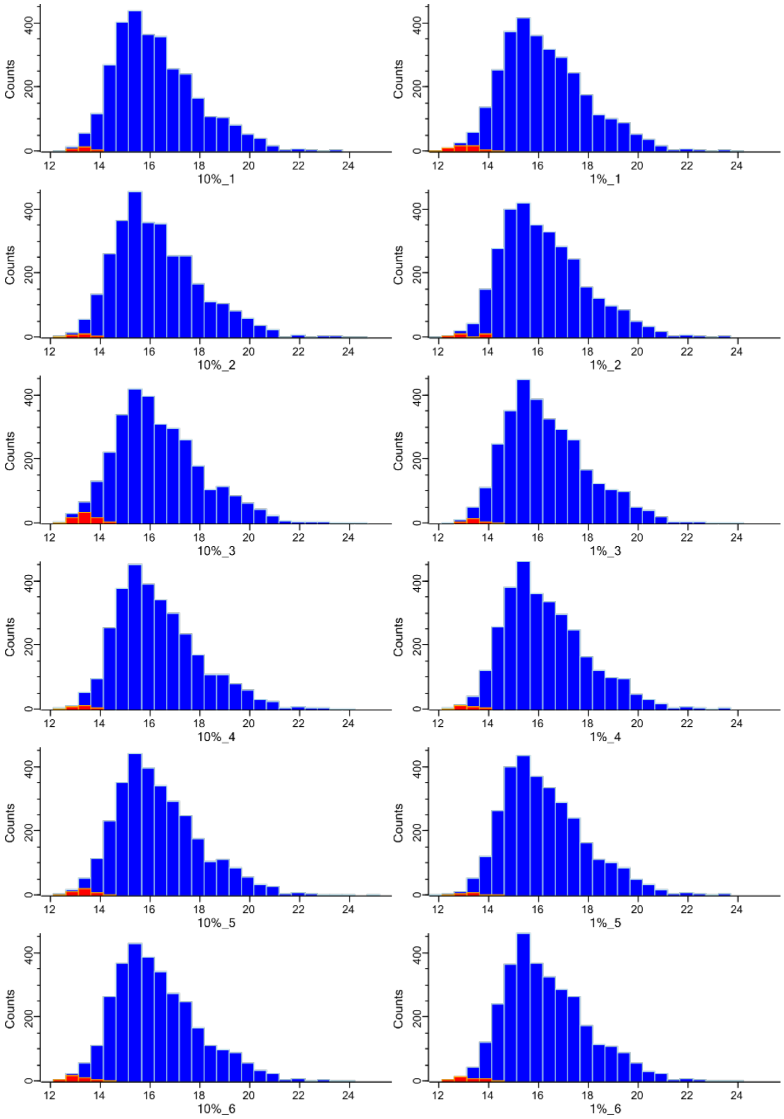

Figure S3. Histograms of LX-2 proteomics imputed values. Left: activated LX-2 cells (10 % FBS). Right: quiescent cells (1 % FBS). Blue bars are measured values, bars in red indicate imputed values (width: 0.3, downshift: 1.8).

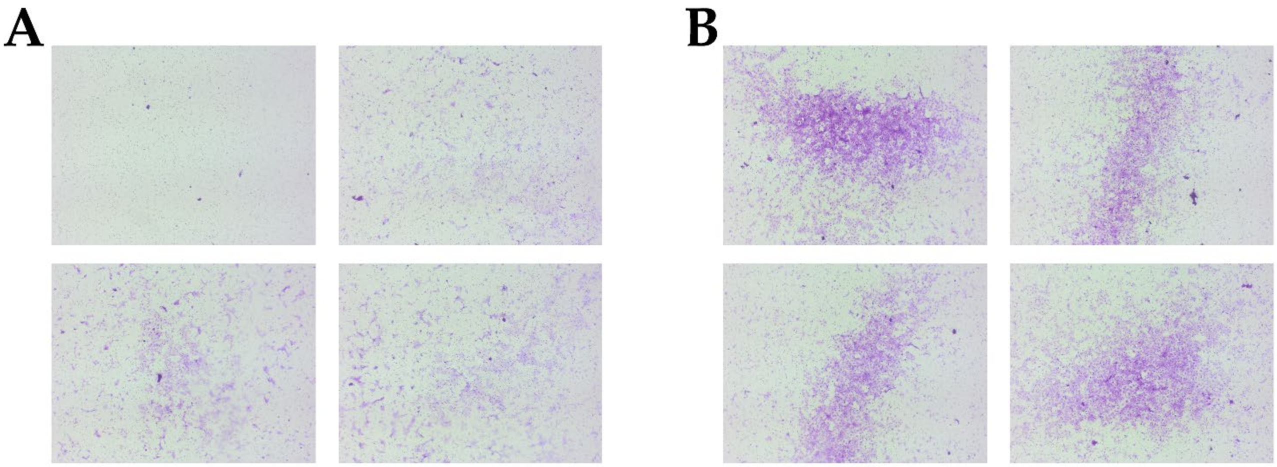

Figure S4. Additional images of LX-2 Transwell migration after 24h. Cells are stained with crystal violet. (**A**) Migration of LX-2 cells in 1 % FBS. (**B**) Migration of LX-2 cells in 10 % FBS.
